# Real-Time Parking Space Detection Based on Deep Learning and Panoramic Images

**DOI:** 10.3390/s25206449

**Published:** 2025-10-18

**Authors:** Wu Wei, Hongyang Chen, Jiayuan Gong, Kai Che, Wenbo Ren, Bin Zhang

**Affiliations:** 1 School of Intelligent Connected Vehicle, Hubei University of Automotive Technology, Shiyan 442002, China; 202311313@huat.edu.cn; 2 Hanjiang National Laboratory, Wuhan 430000, China; dr.chenhongyang@outlook.com; 3 Hubei Provincial Engineering (Technology) Research Center of Automotive Intelligent Networking and Electronic Control, Hubei University of Automotive Technology, Shiyan 442002, China; 4 International Joint Research Center of Automotive Cloud Computing and Simulation Control, Hubei University of Automotive Technology, Shiyan 442002, China; 5 Air-Ground Crowd Cooperation Key Shiyan Laboratory, Hubei University of Automotive Technology, Shiyan 442002, China; 6 School of Optoelectronic Science and Engineering, University of Electronic Science and Technology of China, Chengdu 611731, China; chekaiyc@std.uestc.edu.cn; 7 School of Mechanical and Automotive Engineering, Science and Technology College of Hubei University of Arts and Science, Xiangyang 441025, China; 202111205@huat.edu.cn; 8 Department of Computer, City University of Hong Kong, Hong Kong 999077, China; bzhang48-c@my.cityu.edu.hk; 9 School of Remote Sensing and Information Engineering, Wuhan University, Wuhan 430000, China; 10 Information Network Center, School of Information Network Security, Xinjiang University of Political Science and Law, Tumxuk 843900, China

**Keywords:** autonomous parking, parking space detection, unstructured environments, panoramic image, environmental perception

## Abstract

In the domain of automatic parking systems, parking space detection and localization represent fundamental challenges that must be addressed. As a core research focus within the field of intelligent automatic parking, they constitute the essential prerequisite for the realization of fully autonomous parking. Accurate and effective detection of parking spaces is still the core problem that needs to be solved in automatic parking systems. In this study, building upon existing public parking space datasets, a comprehensive panoramic parking space dataset named PSEX (Parking Slot Extended) with complex environmental diversity was constructed by integrating the concept of GAN (Generative Adversarial Network)-based image style transfer. Meanwhile, an improved algorithm based on PP-Yoloe (Paddle-Paddle Yoloe) is used to detect the state (free or occupied) and angle (T-shaped or L-shaped) of the parking space in real-time. For the many and small labels of the parking space, the ResSpp in it is replaced by the ResSimSppf module, the SimSppf structure is introduced at the neck end, and Silu is replaced by Relu in the basic structure of the CBS (Conv-BN-SiLU), and finally an auxiliary detector head is added at the prediction head. Experimental results show that the proposed SimSppf_mepre-Yoloe model achieves an average improvement of 4.5% in mAP50 and 2.95% in mAP50:95 over the baseline PP-Yoloe across various parking space detection tasks. In terms of efficiency, the model maintains comparable inference latency with the baseline, reaching up to 33.7 FPS on the Jetson AGX Xavier platform under TensorRT optimization. And the improved enhancement algorithm can greatly enrich the diversity of parking space data. These results demonstrate that the proposed model achieves a better balance between detection accuracy and real-time performance, making it suitable for deployment in intelligent vehicle and robotic perception systems.

## 1. Introduction

The rapid advancement of robotics and intelligent control has driven progress in autonomous perception and decision-making, enabling robots to operate effectively in complex environments [1]. Multi-robot systems further highlight the importance of coordination, navigation, and path optimization, which are also core challenges in autonomous driving [2]. Meanwhile, metaheuristic algorithms provide efficient solutions for perception and path planning problems with high dimensionality and nonlinearity [3,4]. However, existing perception methods in autonomous systems still face limitations in complex, dynamic, or low-visibility conditions, making it difficult to ensure stable and accurate environmental understanding. Consequently, autonomous driving continues to face significant challenges in achieving reliable and precise perception for tasks.

Driverless technology has emerged as a major trend in the development of next-generation automobiles, among which automatic parking represents one of the core enabling technologies [5]. In recent years, with the maturity of LiDAR, cameras, millimeter-wave radar, and other sensor technologies, parking assistance systems have been widely used. The foundation for the development of an automatic parking system has been provided. As the last step in solving the parking problem, automatic parking technology has attracted the research interest of many scholars. In the development of automated parking systems, one of the key issues to be addressed is effective parking space detection.

Parking space detection and localization are mainly divided into two categories based on sensors and computer vision [6]. Sensor-based approaches identify vacant parking spaces by detecting gaps between adjacent vehicles and surrounding obstacles, typically using ultrasonic sensors [7], radar, or LiDAR [8,9]. Visual parking space detection uses methods such as edge detection, template matching, and feature matching to extract feature points and lines to achieve parking space information matching by capturing surrounding image information [10].

Based on the principle of sensor-based ranging, Pohl et al. [11] developed a semi-automatic parking assistance system that integrates the vehicle’s built-in sensors with additional components. Ultrasonic sensors were employed to continuously measure distances between the vehicle and its surroundings in order to detect available parking spaces. However, because the vehicle’s turning angle is not a standard right angle, the reflective properties of sound waves introduce considerable measurement errors at the vehicle’s corners. Then Pelaez et al. [12] explored a method to assist drivers in parking by processing data obtained from the three-dimensional time of flight (ToF) cameras and reconstructing objects around the vehicle. This method solves the problem of performance degradation in bright ambient light (mainly occurring in outdoor parking lots), resulting in shadows and brightness in the image, as well as limited detection of low-reflection objects such as dark cars. The use of sensors for detection requires high detection conditions and reference to obstacles. Secondly, the propagation of signals emitted is easily affected by the environment, leading to inaccurate measurements and ultimately affecting the results of parking space detection.

Computer vision-based detection method: using an onboard camera to capture images around the vehicle body, identify parking space identification lines, and classify them. Finally, in the management module, partition the recognition results and calculate the parking space status (occupied, idle, parking space angle, etc.) for each partition according to the already set partition [13,14]. Wang et al. [15] used four fisheye cameras to build a bird’s-eye view vision system and used Radon straight line detection on the omnidirectional bird’s-eye view stitched by the fisheye cameras to determine the parking space angle by intersecting the detected straight lines. In 2020, Suhr et al. [16] used a Convolutional Neural Network (CNN) to extract the global and local information of the parking space to achieve single-stage detection of the parking space. The literature [17] studied deep learning for parking space detection and applied recurrent neural networks based on an embedded platform to process parking lot images but did not study in depth the output of parking space recognition and empty parking space detection information. The visual detection method is friendly to human intuition and easy to observe and understand. However, it has poor generalization ability and a strong sense of fragmentation in complex environments.

In terms of computational speed and accuracy, computer vision based on deep learning far surpasses traditional detection algorithms based on artificial features and sensors. Therefore, this study uses an in-vehicle AVM (360-degree panoramic imaging) system technology to collect parking spaces and combines deep learning algorithms to enhance the dataset and recognition. This can fully utilize vehicle equipment and improve accuracy and real-time performance in complex environments.

In this study, we propose a complex parking scene parking space detection network based on panoramic images and PP-Yoloe improvement to address the shortcomings of previous research work.

The main contributions of this study are as follows:

- To address the limitations of incomplete datasets under complex conditions (fog, snow, sandstorms, and rain), we construct a diverse parking space dataset, PSEX, by incorporating image depth information and a GAN.
- To enhance the contrast of parking space images in complex environments, a Style Attention Module (SANet) is integrated into the GAN framework.
- Furthermore, an end-to-end improved PP-Yoloe model is proposed for parking space detection in complex scenes, aiming to overcome the shortcomings of existing two-stage approaches and their limited accuracy. Compared with the baseline PP-Yoloe, the proposed method achieves notable improvements in both detection speed and accuracy.

### 1.1. Related Works

#### 1.1.1. Data Processing

It is well recognized that the learning capability of neural networks largely depends on the quality of the input data. Accordingly, appropriate preprocessing and annotation of datasets are essential for facilitating effective feature learning and extraction. Therefore, the size and diversity of the dataset have become very important. The publicly available parking space dataset PS2.0, released by Tongji University in 2018, comprises 12,165 surround-view images of 600 × 600 pixels, each corresponding to a 10 m × 10 m physical area. Among them, 9827 images are designated for training and 2338 for testing, with the test set further divided into six categories: indoor, outdoor-daylight, outdoor-street-light, outdoor-shadow, outdoor-rain, and inclined. However, PS2.0 is limited to parking space detection and does not provide information on occupancy status. Subsequently, in 2018, Tongji University accomplished a hybrid approach combining CNNs (Convolutional Neural Networks) with conventional image processing techniques for parking slot marking segmentation, utilizing their self-collected PSV (Panoramic Surround View) dataset [18]. The publicly released PSV dataset comprises over 4200 panoramic surround-view images, encompassing diverse illumination conditions and multi-category parking slot scenarios.

However, neither the PS2.0 nor the PSV datasets contain data in complex extreme environments. Moreover, these data are processed with data augmentation operators and techniques such as flipping, coloring, cropping, Gaussian noise, Mosai, and Mixup, which are good to improve the diversity of the dataset, but only simple processing of the images increases the number of data samples and does not improve the robustness of the model in complex environments.

Therefore, in response to the shortcomings of existing parking space datasets and traditional data enhancement methods, inspired by adversarial neural network algorithms, we added images of fog, snow, rain, sandstorms, and other scenes to the original image processing of parking spaces in different scenes and states. In order to increase the contrast of parking spaces in complex environments and make them closer to the real environment, we reconstructed the adversarial neural network algorithm LapStyle and combined it with the style attention module to process the dataset.

#### 1.1.2. Detection Algorithm

To improve parking space detection under various lighting conditions, Nguyen et al. [19] proposed mAlexNet, specifically designed for smart cameras to detect parking space occupancy, which is a deep convolutional neural network (DCNN) applied to parking lot surveillance images for the first time. However, this method is only applicable to parking lot surveillance images and cannot fully meet the demand of parking space detection. Subsequently, Zhang et al. [20] from Tongji University proposed the DeepPS method, which uses the DCNN technique to detect parking spaces, the YOLO algorithm to detect marker points, and the classification network to obtain parking space information (including the features of parking slots). However, the method is sensitive to changes in parking line direction, has insufficient generalization capability, and has a poor ability to adapt to different scenarios.

Therefore, the PP-Yoloe algorithm [21] has been improved by using the SimSPPF module and adding detection auxiliary heads. Distributed training has been used to integrate parking space detection into a single problem. The parking space angle (T-shaped, L-shaped) and parking space status (vertical idle, vertical occupied, parallel idle, parallel occupied) are detected in the panoramic image at once, achieving end-to-end fast detection results. At the same time, an enhanced version of the dataset combined with adversarial neural networks is used to better cope with parking space detection under extreme weather conditions. The ultimate goal is to achieve fast end-to-end and accurate positioning and detection of parking spaces.

## 2. Materials and Methods

### 2.1. Data Augmentation

Data augmentation is a common method used in target detection and deep learning to improve model performance. Its main purpose is to make the model more generalizable by increasing the diversity of the training dataset. Common forms of data augmentation include methods such as random cropping, level flipping, and color enhancement [22], which improve the robustness of the model to panning, reflection, and illumination conditions, respectively. In addition, methods such as random scaling [23], random rotation, and affine transformation are sometimes used.

**Figure 1:**
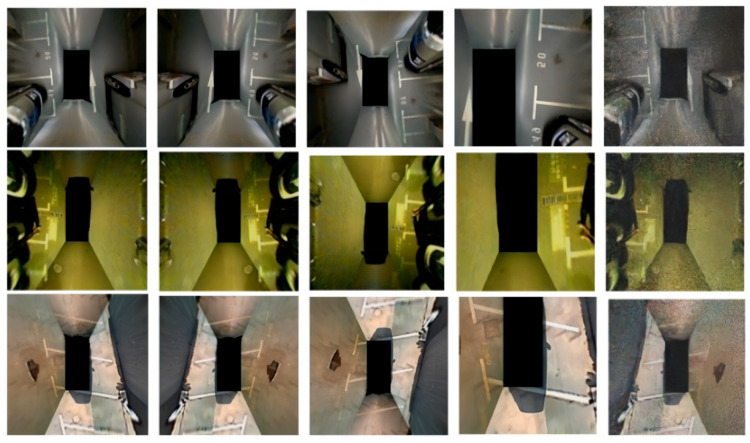
Data augmentation: flip, crop, noise (sort by column).

As shown in Figure 1, common data enhancement approaches can expand the number of datasets to some extent by making some changes to the images and adding and calling data augmentation operators to the neural network structure to add changes to each set of batch data to ensure that the network can learn more feature information. However, these methods only perform shallow operations on the image and do not make the image contain more information about the environmental features. For example, classical target detection algorithms such as YOLOv4 [24] and YOLOv5 use data augmentation operators (mosaic, augment_hsv, flip) for data augmentation.

Therefore, to address the limitations of the model in handling detection tasks in more complex environments, we were inspired by generative adversarial neural networks (GAN) [25] and used style migration techniques to fuse different extreme environments, such as rain, snow, and fog, into the original image without changing the original image information, to achieve foggy, rainy, and snowy car parking image generation. This fused image is very close to the real scene. By style migration of GAN, data no longer just changes the information of the surface layer of the image but also augments the data in terms of deeper image semantics and environment style, thus increasing the size of the dataset and adding more different semantic and feature information, which greatly improves the generalization ability of the trained model.

### 2.2. Data Augmentation Algorithm for Style Transfer

Image style migration is an image processing method that renders the semantic content of an image in a different style [26]. Traditional non-parametric style migration methods can only perform texture synthesis by extracting low-level features (color, texture, etc.) of an image and cannot extract higher-level features of an image. Efros et al. [27] synthesized the target image by extracting and reorganizing texture samples; Hertzman et al. [28] transformed existing image styles to the target image by image analogy; Ashikhmin [29] transformed the high-frequency texture of the source image to the target image while preserving the coarse scale of the target image; Lee et al. [30] enhanced this algorithm by passing additional edge information. Although the traditional nonparametric methods achieve some results, they all have limitations: they only extract low-level features of the target image.

The neural network has excellent feature extraction ability and can capture rich semantic information, which is the foundation on which style migration relies. With algorithms such as DualGAN, CyCleGAN, Pix2Pix, and SSIM-GAN being proposed one by one, style migration techniques become mature. For example, the Multi-Content GAN [31] project at Berkeley’s BAIR Lab, in collaboration with Adobe in 2018, proposed a leave-one-out training method that uses only a small number of letters to generate mostly unseen letters and can replicate the colors and textures of the original samples. In 2019, Peking University and Adobe Research Institute proposed ShapeMatchingGAN [32], which achieves a more concise rendering of artistic letter styles. The network uses a bidirectional shape matching and forward or reverse structure migration strategy to train on just one user-specified style image and supports controllable text styles.

The first publicly available parking space dataset, PS2.0, contains only six types of parking spaces: “indoor”, “outdoor daylight”, “outdoor street light”, “outdoor shade”, “outdoor rain”, and “tilt”, followed by the subsequent public PSV parking dataset, which only classifies the status of parking spaces in PS2.0. The second and subsequent publicly available PSV parking datasets only classify the status of parking spaces in PS2.0. Neither of these datasets includes data on parking spaces in complex and extreme environments. Therefore, to increase the diversity of the parking space dataset, the idea of style migration is used to process the images.

For the algorithm, the choice is made to use the LapStyle [33] algorithm in PaddleGAN. The algorithm first transmits a low-resolution global style pattern through the drawing network and then performs high-resolution correction of local details through the correction network. The correction network generates residual images using the draft and Laplace-filtered extracted image textures. By stacking multiple correction networks and Laplace pyramid levels, higher-resolution details can be generated more easily. Ultimately, the stylized image is obtained by aggregating the output of all pyramid levels.

**Figure 2:**
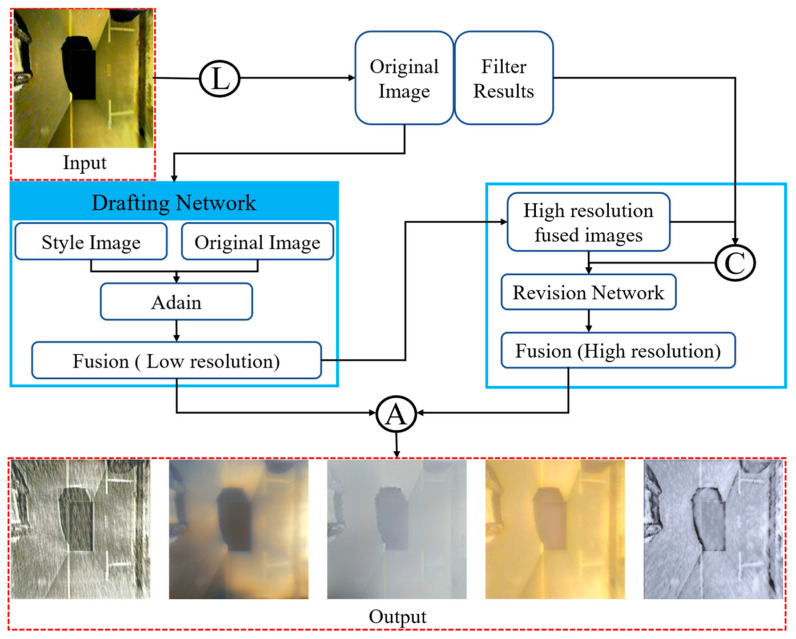
LapStyle algorithm process.

Figure 2 illustrates the flow of the LapStyle algorithm, where L, C, and A represent Laplace, connection, and aggregation operations.

First, the Drafting Network is utilized to transfer low-resolution global style patterns. This network fuses the original image and the style image in low-resolution mode and transmits the fusion result to the Revision Network after 2× upsampling. In the Revision Network, we use high resolution for continuous correction. Finally, the corrected image is operated with the low-resolution fused image for aggregation to generate a fused image with true style. The algorithmic design was inspired by the SANet [34] network algorithm. In the LapStyle structure, it is planned to include a style-focused module. The style attention module is a module commonly used for computer vision tasks that can guide the neural network to pay more attention to the style information of images, thus improving the performance and effectiveness of the model.

Specifically, the style attention module can separate the content and style information of an image and process them differently. By enhancing the stylistic information in the image, the model can pay more attention to the details of the image, such as texture, color, shape, etc. Thus enhancing the clarity and realism of the image. In addition, the structure and classification of the PS2.0 dataset are analyzed in depth. As the added environment is not found in real indoor scenes, the composition of the five synthetic styles (‘outdoor daylight,’ ‘outdoor street light,’ ‘outdoor shadow,’ ‘outdoor rain,’ and ‘tilt’) is presented. These styles were generated in a ratio of about 1:10, with the detailed composition provided in Table 1.

After the data enhancement by the GAN method, the fog, sandstorm, snowstorm, and rain scenes were fused with each type of the original PS2.0 to obtain the enhanced dataset, and the number of various types was increased by nearly 1000% compared with the original dataset, such as the outdoor daylight type from 546 to 5552, and the number was increased at the same time to increase the diversity of car parking information. We named the augmented dataset PSEX, and it consists of images selected from the original PS2.0 dataset, with resolutions of 512 × 512, 256 × 256, and 128 × 128. After the augmentation of the train part, the training data can be greatly increased, which is key to improving the amount of data at the same time, and also greatly enriches the feature information of the parking space. Provide solid conditions for subsequent network training.

### 2.3. Dataset Validation

In order to evaluate the effect of data enhancement, in this paper, 3000 images are randomly selected from the 6000 generated images and form a new dataset with 3000 randomly selected images from the original dataset, totaling 6000 images. This dataset is divided into 4800 images for the training set and 1200 images for the testing set. At the same time, this paper also randomly selects the same number of pictures from the original dataset to form a comparison group, which contains a small number of pictures of complex environments, and the control group is divided into a training set in the same way as above. This paper employs three object detection models—YOLOv8, YOLOx, and Fast R-CNN—for experimental evaluation. All models were trained and evaluated under a consistent protocol, using the same learning rate, optimizer, and, crucially, an identical test set to ensure a fair comparison. The evaluation is based on the mean Average Precision (mAP) and mean Recall across all categories.

From Table 2, the results demonstrate that the GAN-based data augmentation method effectively enhances both AP (Average Precision) and Recall rates under consistent data volumes. This improvement strengthens the model’s generalization capability and robustness while also boosting parking space detection accuracy across diverse environmental conditions.

### 2.4. Parking Space Detection Algorithm

**Figure 3:**
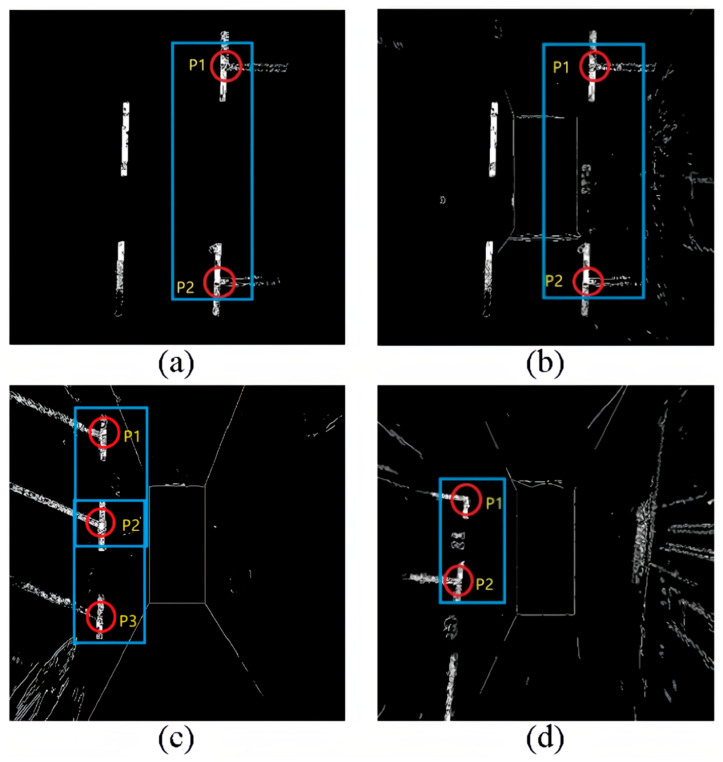
Different types of cars, parking corners, and marking lines. (**a**) Schematic diagram of parking space header; (**b**) Horizontal parking space; (**c**) Angled parking space; (**d**) Perpendicular parking space.

Parking space detection is mainly divided into two parts: parking space type and parking space status detection. The type of parking space is mainly determined by the front of the parking space, which usually includes paired marking points. As shown in Figure 3.

Figure 3 shows different types of parking space headers and marking lines. These four images are binarized from the original image to highlight the marking lines. We analyze the parking space. A parking space consists of four points; two points far from the body are generally not observed, but in the usual parking process, we also only need to observe the two closest parking points to the body to park. That is to say, the type of detection of a parking space is mainly to detect the two points closest to the car itself, such as P1, P2, and P3 in the figures. The combination of P1, P2, and P3 is also called the head of the parking space. And each of these parking heads has different shapes, which we define as “T” and “L” shapes, respectively. Since we are analyzing and processing based on the panoramic parking image and the AVM (360-degree panoramic image) used by all major car manufacturers nowadays to display visual information, when recognizing parking spaces, only the two parking points closest to the body need to be detected, and we do not need to additionally reason about the remaining two parking points and just go directly to detect the parking corners and parking spaces.

PSD_L [35] and DeepPS are two representative marker point-based approaches, where PSD_L uses a machine learning-based detection scheme to detect marker points and DeepPS uses a dcnn-based object detection framework to detect marker points. Although both methods are effective in detecting various parking spaces, they both require a complex rule-based scheme or time-consuming local image classification to match pairs of entry line marker points, which leads to a tedious process of inferring complete parking spaces.

**Figure 4:**
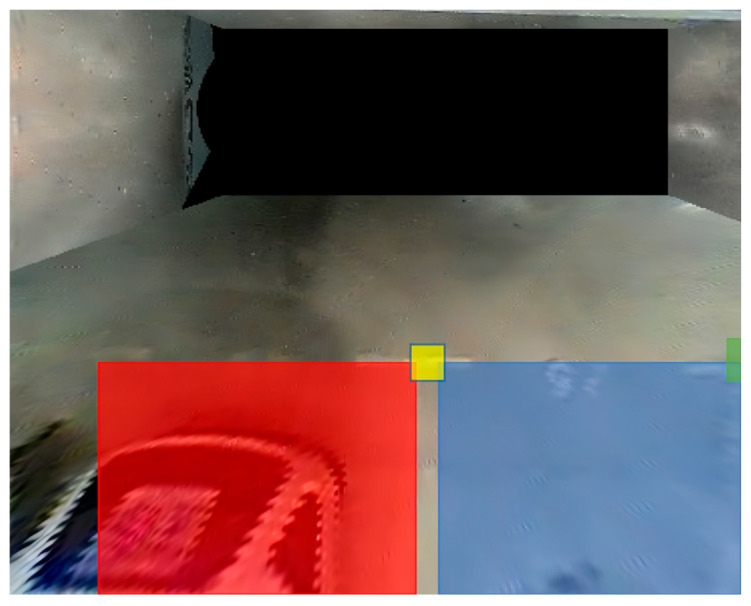
Labeling of different car parking angles.

In contrast, combining header detection and parking space corner categorization into one task allows for quick and easy detection of various parking space corners. Examples include the previously defined “T” and “L” shaped corners. To quickly detect and recognize parking space corner types, target detection methods are used, and although there are many deep learning-based target detection methods, they can be divided into two categories: one-stage [36,37,38] and two-stage detection [39,40]. Considering the real-time and detection speed requirements of parking space detection, PP-Yoloe was selected, which is a method based on an algorithm improved from the YOLOv3. To train the recognition of the parking space angle detector, P1 and P2 labels were made on the parking space. Dividing them into “T_corner” and “L_corner” labels as in Figure 4. The green box represents “L_corner”, and the yellow box represents “T_corner”.

After the corner labeling and detection, there is still one part left for the parking space detection: the parking space status detection. The parking space status is mainly divided into two kinds: idle and occupied. In the parking space corner labeling, we are also labeling the parking space status for the panoramic parking space image detection.

**Figure 5:**
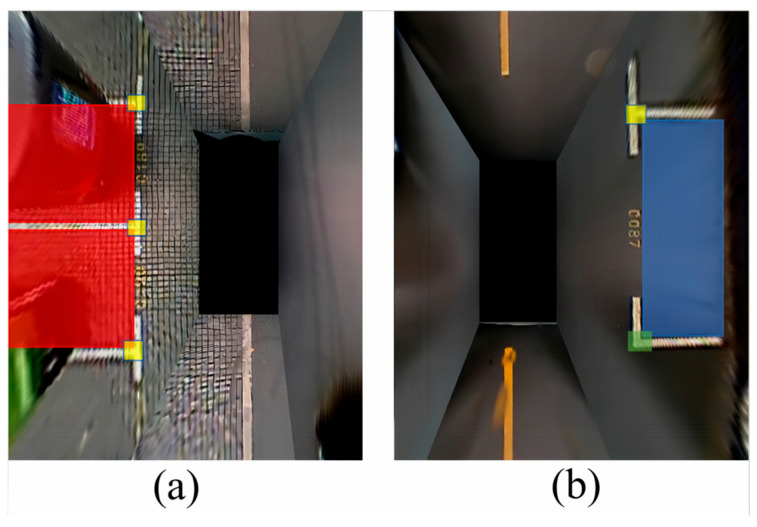
Car parking status marker. (**a**) Parallel occupied parking spaces; (**b**) Parallel idle parking spaces.

Figure 5 shows how the parking space status is labeled, with the red box in (a) representing parallel occupied spaces and the blue box in (b) representing parallel idle spaces. The red and blue boxes in Figure 4 represent vertical occupied spaces and vertical free spaces. The parallel and vertical are defined relative to the vehicle body in the panoramic image, which is also in line with the driver’s intuition.

**Figure 6:**
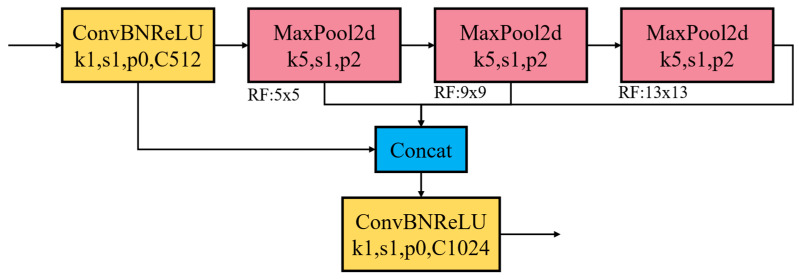
SimSPPF structure.

In the algorithmic implementation process, this study conducted multi-dimensional structural improvements and computational efficiency optimization for the PP-Yoloe model. Given that parking slot detection is the core perception task in autonomous parking systems, the model must strictly satisfy the dual constraints of accuracy and robustness and real-time performance. Inspired by the SPPF module in YOLOv6 [41], this study upgraded the native SPP structure of PP-Yoloe to SimSPPF, whose architecture is illustrated in Figure 6. The key improvement lies in reconstructing the original parallel multi-branch max-pooling operations of SPP into a single-branch multi-level cascaded structure. Specifically, the SPP kernel sizes are 5 × 5, 9 × 9, and 13 × 13, whereas all pooling layers in SimSPPF adopt a unified kernel size of 5 × 5. Equations (1) and (2) are employed to dynamically adjust pooling parameters to ensure consistent output feature map dimensions while minimizing kernel redundancy.

(1) $$\begin{array}{l} K_{h}=ceil(\frac{h_{in}}{n})\\ p_{h}=floor(\frac{K_{h}\cdot n-h_{in}+1}{2})\\ h_{new}=2p_{h}+h_{in} \end{array}$$

where $h_{in}$ is the original height of the input feature map, n is the target output dimension, $K_{h}$ is the actual height of the pooling kernel, calculated from the input and target dimensions, $p_{h}$ is the padding amount applied to the height dimension, $h_{new}$ is the adjusted input height after padding, ceil is the Ceiling function (rounding upward) and the floor is the Floor function (rounding downward).

(2) $$\begin{array}{l} K_{w}=ceil(\frac{w_{in}}{n})\\ p_{w}=floor(\frac{K_{w}\cdot n-w_{in}+1}{2})\\ w_{new}=2p_{w}+w_{in} \end{array}$$

where $w_{in}$ is the original width of the input feature map, $K_{w}$ is the actual width of the pooling kernel, calculated from the input and target dimensions, $p_{w}$ is the padding amount applied to the width dimension and $w_{new}$ is the adjusted input width after padding.

Assuming the target output dimension is n, the input channel count is W, and the input size is $w_{in}\times h_{in}$, the computational complexity of the SimSPPF module is significantly reduced through parameter optimization using Equations (1) and (2), as demonstrated in Equation (3):

(3) 3×(5×5)×W<(5×5+9×9+13×13)×W

Furthermore, to further enhance the model inference efficiency, considering the hardware-friendly nature of ReLU that enables more efficient fixed-point computation on GPUs/FPGAs, the SiLU activation function in the CBS (Conv-BN-SiLU) module is replaced with ReLU (the improved module is named CBR).

**Figure 7:**
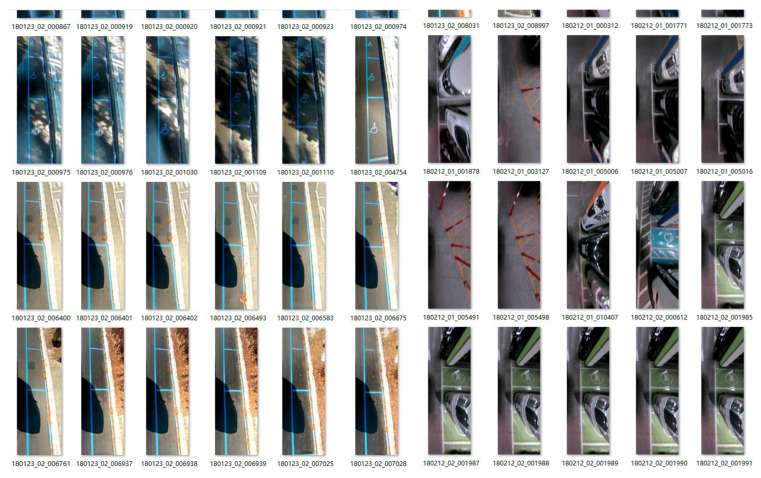
PIL_PARK dataset.

Secondly, since the panoramic image of the vehicle position contains such small targets similar to “T_corner” and “L_corner”, three SimSPPF modules are added directly to the P5, P4, and P3 layers of the neck structure output of the network with the Head structure. The fixed-length feature vectors generated by the SimSPPF structure can be fused with other feature maps in the neck to obtain more expressive feature vectors. During the training process, it is noticed that the selection of the pre-trained model is also highly related to the learning ability and learning time of the model. Therefore, another vehicle location group dataset was selected, PIL_PARK, as shown in Figure 7.

The same labeling method as PS2.0 is adopted for the PIL_PARK dataset, and this pre-trained model is loaded during the training phase to allow the network to converge quickly. Finally, since the target scale for the detection of the parking spaces spans too large, from the corner type to the state type, it takes a lot of time to identify. Thus, a detection head is added to allow the model to simultaneously use different levels of feature maps for target detection, and it allows for better learning and optimization of target objects at different scales separately, improving the detection accuracy and robustness of the model.

**Figure 8:**
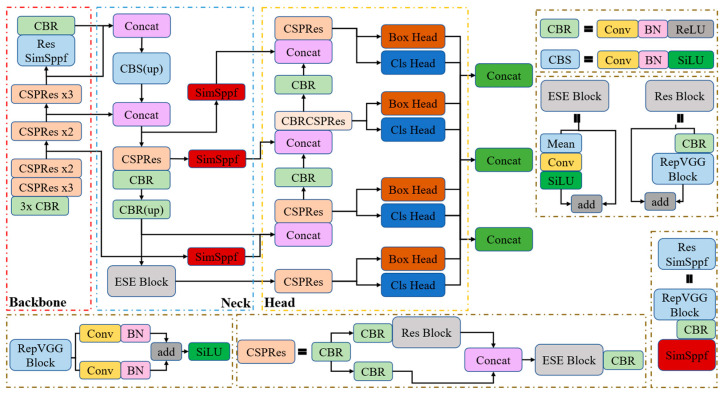
Structure diagram of the improved PP-Yoloe model.

In summary, as shown in Figure 8, the model network design is divided into three parts: the backbone part for feature extraction, the neck part for semantic representation of extracted features, and the head part for classification and prediction. The main improvement: replacing ResSpp with the ResSimSppf module in the backbone network, introducing the SimSppf structure at the neck end, replacing Silu with Relu in the basic CBS structure, and finally adding the auxiliary detection head at the prediction end.

## 3. Results and Discussion

To validate the method proposed in this article, we utilized an Intel Core i7-7700 CPU, two GPUs with 8 GB of memory each (GTX2080), CUDA 11.6 for GPU acceleration, OpenCV 4.5.1 for the computer vision library, and the PaddlePaddle deep learning framework.

### 3.1. Data Augmentation Section

In the third stage, a GAN algorithm is used, and a photo of a natural landscape is selected. We conducted three rounds of training with different resolutions: low-resolution of 128 × 128, 256 × 256 and high-resolution of 512 × 512. The initial learning rate for the epoch was set to 1 × 10^−4^. We set the content_weight to 1.0 and style_weight to 3.0. Each round consisted of 30,000 iterations. To achieve desirable results, the large-scale coco2017 dataset was used as a reference for training.

**Figure 9:**
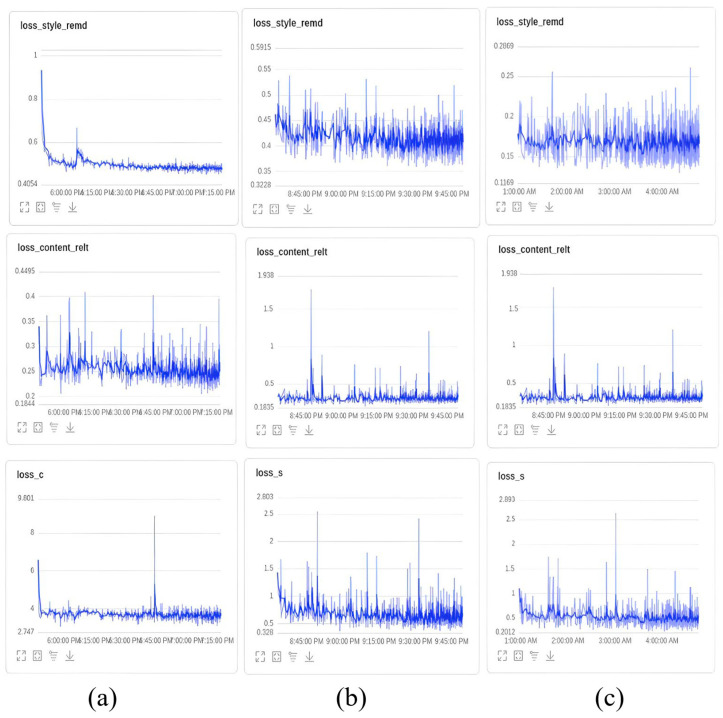
GAN-style migration train loss. (**a**) Training loss curve at 128 × 128 resolution; (**b**) Training loss curve at 256 × 256 resolution; (**c**) Training loss curve at 512 × 512 resolution.

**Figure 10:**
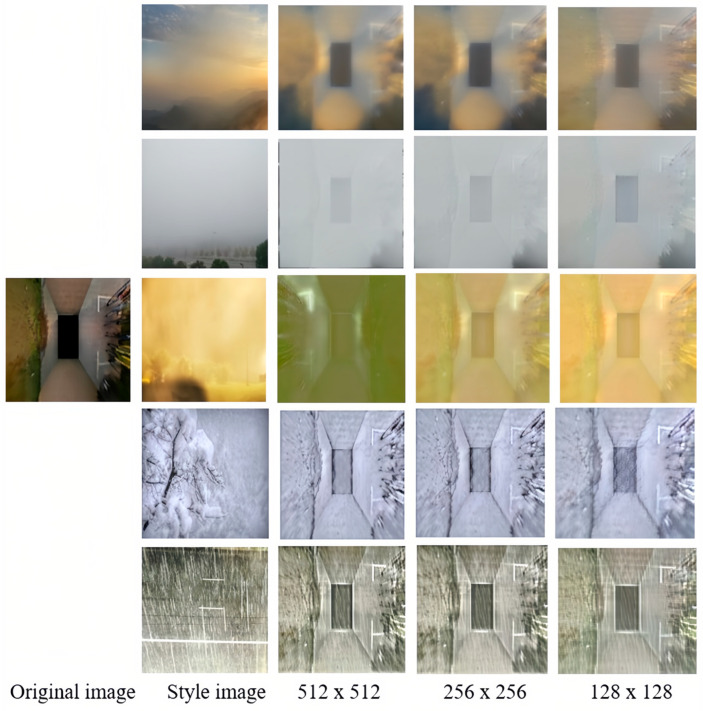
Effect of data augmentation.

Figure 9 illustrates the loss during the three training rounds for 128 × 128 (Figure 9a), 256 × 256 (Figure 9b), and 512 × 512 (Figure 9c) resolutions. It can be observed that the content-related loss function (loss_content_relt) steadily decreases in each round, reaching a final value of 0.1835. This indicates an increasing correlation between the feature representations of the input and target images at various levels. Similarly, the style transfer difference loss function (loss_style_remd) also decreases continuously, indicating a decreasing difference between the feature representations of the input and target images at different levels. Additionally, the losses (loss_c and loss_s) demonstrate that the differences between the content and style features of the input and target images diminish as well. Figure 10 showcases the transformed images of a parking lot’s original image using five different styles, generating distinct feature maps of various sizes.

### 3.2. Parking Space Recognition and Detection Algorithms

**Figure 11:**
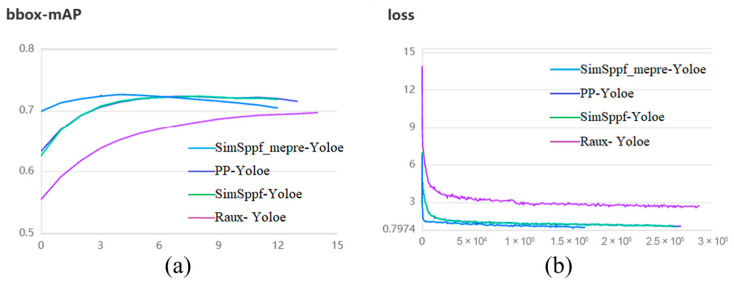
Test set scores and training loss curves for each model according to mAP values. (**a**) mAP-based testing scores of each model; (**b**) Training loss curves of each model.

To evaluate the neural network’s capabilities, we randomly divided the dataset in an 8:2 ratio, with 80% for training and 20% for testing. The testing data was kept separate from the training process to objectively assess the training results. In our experiments, the training iterations were set to 300, the initial learning rate to 0.00125, and the Adam optimizer was utilized. The network was fed with four images at once, with an input size of 640 × 640. Building upon the improvements from the third phase, we constructed three network models: SimSppf-Yoloe (+SimSppf), SimSppf_mepre-Yoloe (+SimSppf+PIL_PARK, pretraining weights), and Raux-Yoloe (+SimSppf+auxiliary detection head+small input size). These three models were compared with the baseline PP-Yoloe on the same augmented dataset. Figure 11 displays the testing scores based on mAP values and training loss curves for each model. To ensure fair comparison, the epoch is set to 300. It can be observed that although the mAP value for SimSppf_mepre-Yoloe starts to decline at step 6, it consistently exceeds 0.7 from the beginning of training, significantly surpassing the original PP-Yoloe algorithm. Moreover, it reaches convergence by Step 6, indicating that the convergence time of the model is accelerated after incorporating the pretrained weights from the PIL_PARK dataset. This observation is also evident from the loss curve in (Figure 11b).

**Figure 12:**
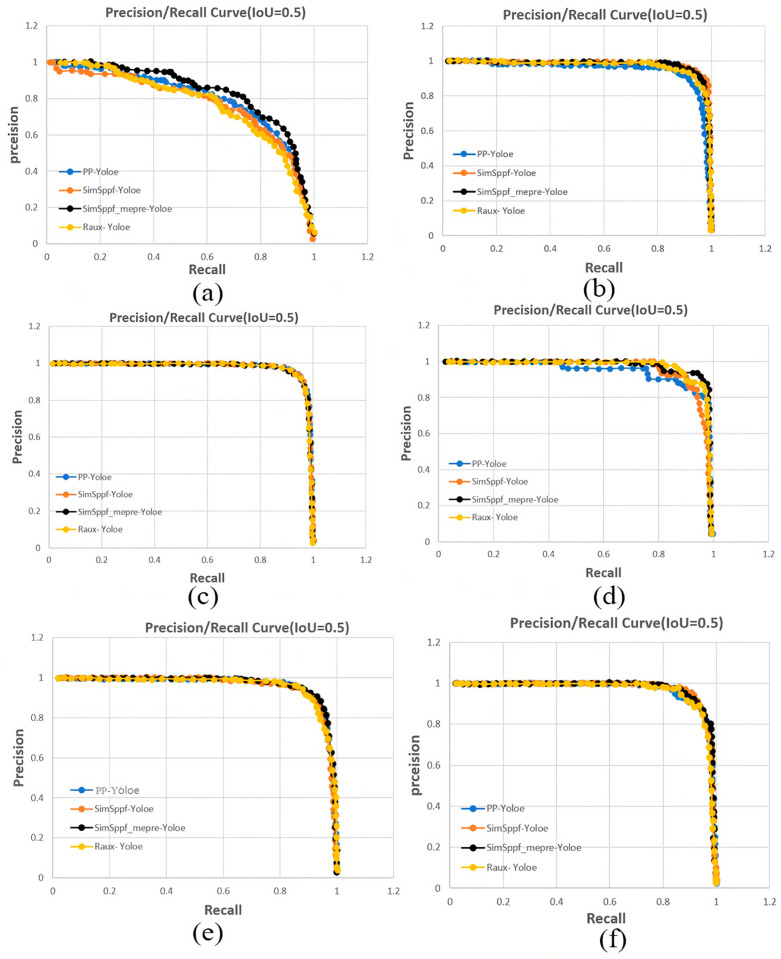
The PR curves for detecting parking space corners and parking space occupancy status using different algorithms. (**a**) PR curve of L_corner category; (**b**) PR curve of Parallel_parking_freespace category; (**c**) PR curve of Parallel_parking_occupancyspace category; (**d**) PR curve of T_corner category; (**e**) PR curve of Vertical_parking_freespace category; (**f**) PR curve of Vertical_parking_occupancyspace category.

Furthermore, the experiments were conducted using all three models to detect the angle and state of parking spaces. Figure 12 illustrates the PR (precision-recall) results for each label category.

**Figure 13:**
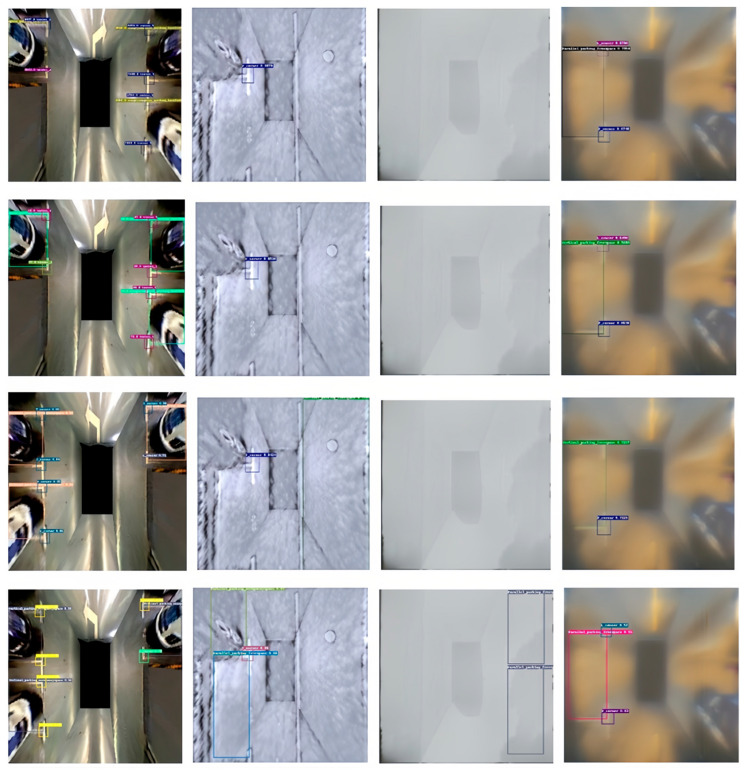
Testing of the four algorithms, namely PP-Yoloe, SimSppf-Yoloe, Raux-Yoloe, and SimSppf_mepre-Yoloe, on the PS2.0 and PSEX datasets (sorted by column).

We divided the entire parking space dataset into six types: L_corner, Parallel_parking_freespace, Parallel_parking_occupancyspace, T_corner, Vertical_parking_freespace, and Vertical_parking_occupancyspace. As shown in Figure 12a–f, they correspond to the PR curves of these six label categories. It is evident from the graph that the SimSppf_mepre-Yoloe algorithm, represented by the black curve, achieves the best detection performance among the label categories. To further evaluate the performance of the improved algorithm, the PS2.0 and PSEX datasets are tested using four detectors: PP-Yoloe, SimSppf-Yoloe, SimSppf_mepre-Yoloe, and Raux-Yoloe. The test results are shown in Figure 13.

From Figure 13, it can be observed that all four algorithms perform accurately in detecting parking spaces in the original PS2.0 dataset. However, the second column indicates that the PP-Yoloe, SimSppf-Yoloe, and Raux-Yoloe algorithms fail to accurately detect parking spaces in heavy snow conditions. In contrast, the SimSppf_mepre-Yoloe algorithm can accurately detect parking spaces in such conditions and can also identify and detect parking space corners and occupancy status without errors. The third and fourth columns further demonstrate the effectiveness of the SimSppf_mepre-Yoloe algorithm in handling complex environments.

At the same time, under the same equal test set, we evaluated the accuracy (mAP) of different models in detecting various parking space types. Table 3 compares the mAP performance of PP-Yoloe, SimSppf-Yoloe, and the proposed SimSppf_mepre-Yoloe across various parking space detection tasks. The results show that SimSppf_mepre-Yoloe consistently outperforms the baseline PP-Yoloe, achieving an average improvement of approximately 4.5% in mAP50 and 2.95% in mAP50:95. Notably, the detection of parking space occupancy exhibits significant gains, with parallel parking occupancy increasing from 0.732 to 0.793 (mAP50) and from 0.703 to 0.743 (mAP50:95), while vertical parking occupancy rises from 0.766 to 0.789 (mAP50) and 0.691 to 0.711 (mAP50:95). In addition, corner detection also benefits remarkably from the improvements, with T_corner increasing from 0.687 to 0.750 (mAP50) and from 0.633 to 0.674 (mAP50:95), and L_corner improving from 0.457 to 0.545 (mAP50) and from 0.442 to 0.493 (mAP50:95). These results highlight the enhanced capability of the proposed model in capturing fine-grained spatial features. Compared to SimSppf-Yoloe, the proposed model demonstrates further accuracy gains while maintaining a lightweight network structure, indicating its effectiveness and robustness across multiple parking scenarios.

Finally, considering the application of this detection method in automated parking, it is important to consider the size and real-time performance of the network models. Therefore, tests are conducted on Ubuntu, Jetson Nano, and Jetson AGX Xavier platforms. The inference speeds in different operating systems and testing modes are shown in Table 4.

Table 4 presents the inference speeds of four network models—PP-Yoloe, SimSppf-Yoloe, SimSppf_mepre-Yoloe, and Raux-Yoloe—on different computing platforms (Ubuntu PC, Jetson AGX, and Jetson Nano) under various modes (No_trt, Trt_16, and Trt_32). The results indicate that SimSppf_mepre-Yoloe maintains inference efficiency comparable to PP-Yoloe. On the Jetson AGX platform with TensorRT 16-bit optimization, it achieved a latency of 29.7 ms, which corresponds to a frame rate of 33.7 FPS (calculated as 1000/29.7). Raux-Yoloe achieves the highest speed across all platforms, reaching 105.3 FPS (calculated as 1000/9.5) on Jetson AGX, but with a slight trade-off in accuracy.

Experimental results demonstrate that SimSppf_mepre-Yoloe achieves a superior balance between detection accuracy and inference speed. The proposed model achieves an average improvement of 4.5% in mAP50 and 2.95% in mAP50:95 over the baseline PP-Yoloe across multiple parking space detection tasks, while maintaining comparable inference efficiency on edge computing platforms such as Jetson AGX. These findings indicate that SimSppf_mepre-Yoloe outperforms PP-Yoloe in terms of overall performance. Compared to Raux-Yoloe, which achieves higher frame rates at the cost of a slight accuracy degradation, SimSppf_mepre-Yoloe offers a more balanced trade-off, making it better suited for deployment on edge devices in intelligent vehicle and robotic systems.

## 4. Conclusions

This paper addresses the limitations of traditional data augmentation methods and existing parking datasets, which are often limited in variety and lack representation of complex scenarios. To tackle these challenges, we employ GAN neural networks to process parking images and introduce a novel parking dataset called PSEX. This dataset not only expands the scale of existing datasets but also enriches the feature information of parking spaces under challenging environmental conditions.

Furthermore, we enhance the PP-Yoloe algorithm to simultaneously address two detection tasks: identifying parking space corners and determining occupancy status. The SimSPPF module and an auxiliary detection head are incorporated, enabling the model to leverage the augmented dataset while maintaining both high detection accuracy and efficiency. Experimental results show that the improved SimSppf_mepre-Yoloe algorithm achieves an average improvement of 4.5% in mAP50 and 2.95% in mAP50:95 over the baseline PP-Yoloe across various parking detection tasks, including corner and occupancy recognition. In terms of efficiency, the model attains up to 33.7 FPS on the Jetson AGX platform under TensorRT 16-bit optimization, demonstrating a favorable balance between real-time performance and detection precision. Compared with Raux-Yoloe, which reaches higher FPS but at the cost of some accuracy, SimSppf_mepre-Yoloe provides a robust and accurate solution for parking space detection. These results also offer valuable insights for environmental perception research in intelligent vehicles and service robots.

Finally, in order to improve the accuracy and detection speed of the model, model quantization techniques will be used to further reduce the size of the model. Our future work will focus on enhancing the model’s detection speed for deployment on specific edge devices like UAVs (Unmanned Aerial Vehicles) and autonomous driving computing units, aiming to achieve a balance between speed and accuracy in these real-time application scenarios.

## Figures and Tables

**Table 1:** Dataset structure before and after data augmentation.

Type	Original/(Count)	Style Augmentation/(Count)
Indoor-parking-lot	226	226
Outdoor-normal-daylight	546	5552
Outdoor-rainy	244	2464
Outdoor-shadow	1127	11,905
Outdoor-slanted	48	520
Outdoor-street-light	1477	1505
Train	9827	22,637

**Table 2:** Experimental results of dataset validation.

Algorithm Type	Data Type	AP	Recall
YOLOv8	No augmentation	0.78	0.79
	Traditional data augmentation	0.85	0.86
	GAN data augmentation	**0.90**	**0.89**
YOLOx	No augmentation	0.71	0.73
	Traditional data augmentation	0.80	0.82
	GAN data augmentation	**0.82**	**0.83**
Fast R-CNN	No augmentation	0.69	0.66
	Traditional data augmentation	0.72	0.70
	GAN data augmentation	**0.77**	**0.74**

**Table 3:** Comparing mAP scores of different models.

Parking Information	mAP	PP-Yoloe	SimSppf-Yoloe	SimSppf_Mepre-Yoloe	Deviation (%)
Parallel_parking_freespace	mAP50	0.860	0.862	**0.881**	**+2.44%**
	mAP50:95	0.752	0.749	**0.768**	**+2.13%**
Parallel_parking_occupancyspace	mAP50	0.732	0.744	**0.793**	**+8.33%**
	mAP50:95	0.703	0.712	**0.743**	**+5.69%**
Vertical_parking_freespace	mAP50	0.787	0.789	**0.801**	**+1.78%**
	mAP50:95	0.720	0.732	**0.729**	**+1.25%**
Vertical_parking_occupancyspace	mAP50	0.766	0.776	**0.789**	**+3.00%**
	mAP50:95	0.691	0.697	**0.711**	**+2.89%**
T_concer	mAP50	0.687	0.701	**0.750**	**+9.17%**
	mAP50:95	0.633	0.648	**0.674**	**+6.48%**
L_corner	mAP50	0.457	0.467	**0.545**	**+19.26%**
	mAP50:95	0.442	0.483	**0.493**	**+11.54%**

**Table 4:** Inference speeds (in milliseconds) of the four network algorithms in different unit platforms and testing modes.

Computing Unit Platform	Metric	Ubuntu (PC)			Jetson AGX			Jetson Nano		
Different Modes		No_trt	Trt_16	Trt_32	No_trt	Trt_16	Trt_32	No_trt	Trt_16	Trt_32
PP-Yoloe	Latency (ms)	35.2	11.4	28.8	143.4	30.0	80.0	845.0	342.7	-
	FPS	28.4	87.7	34.7	7.0	33.3	12.5	1.2	2.9	-
SimSppf-Yoloe	Latency (ms)	33.1	10.0	27.5	142.7	30.7	80.9	825.0	337.8	-
	FPS	30.2	100.0	36.4	7.0	32.6	12.4	1.2	3.0	-
SimSppf_mepre-Yoloe	Latency (ms)	34.6	11.3	27.0	141.6	29.7	79.5	828.8	337.9	-
	FPS	28.9	88.5	37.0	7.1	33.7	12.6	1.2	3.0	-
Raux-Yoloe	Latency (ms)	6.7	3.3	5.2	83.9	9.5	15.3	350.6	66.4	-
	FPS	149.3	303.0	192.3	11.9	105.3	65.4	2.9	15.1	-

## Data Availability

Data are contained within the article.

## Abbreviations

The following abbreviations are used in this manuscript:

GAN	Generative Adversarial Networks
CNN	Convolutional Neural Network
AVM	Around View Monitor
CNNs	Convolutional Neural Networks
PSV	Panoramic Surround View
DCNN	Deep Convolutional Neural Network
AP	Average Precision
SPP	Spatial Pyramid Pooling
SPPF	Spatial Pyramid Pooling-Fast
SimSPPF	Simplified Spatial Pyramid Pooling-Fast
CBS	Conv-BN-SiLU
CBR	Conv-BN-ReLU
PR	Precision-recall
mAP	Mean Average Precision
